# The mechanism: how dental resorptions occur in ameloblastoma

**DOI:** 10.1590/2177-6709.24.4.021-032.oin

**Published:** 2019

**Authors:** Giovana Gonçalves Martins, Ingrid Araújo de Oliveira, Alberto Consolaro

**Affiliations:** 1 Mestre em Odontopediatria, Universidade de São Paulo, Faculdade de Odontologia de Ribeirão Preto (Ribeirão Preto/SP, Brazil).; 2 Universidade de São Paulo, Faculdade de Odontologia de Ribeirão Preto, Programa de Pós-Graduação em Odontopediatria (Ribeirão Preto/SP, Brazil).; 3 Hospital Infantil Dr. Juvêncio Mattos (São Luís/MA, Brazil).; 4 Hospital Geral Tarquinio Lopes Filho, Serviço de Cirurgia e Traumatologia Bucomaxilofacial (São Luís/MA, Brazil).; 5 Universidade de São Paulo, Faculdade de Odontologia de Bauru (Bauru/SP, Brazil).

**Keywords:** Tooth resorption, Root resorption, Ameloblastoma, Lesions of the jaws, Orthodontics

## Abstract

Knife-edge or blunt root resorptions characterize ameloblastomas and are pathognomonic for this tumor, because they differentiate ameloblastomas from simple bone cysts, odontogenic keratocysts and nasopalatine duct cysts, which do not lead to resorption of involved teeth. Despite the very high frequency and importance of these characteristics for a differential diagnosis, a microscopic examination should also be conducted before defining the diagnosis and the treatment plan for these cases. This paper describes a six-step hypothesis to explain the mechanism by which ameloblastomas promote the characteristic root resorptions found in association with these benign epithelial tumors, which have a fibrous capsule formed by islands and epithelial cords that mimic the dental lamina, invade neighboring tissues and release mediators (IL-1, EGF) of tooth and root resorption. This hypothesis may be one more explanation for the tooth resorptions sometimes found in orthodontic records, and may help differentiate the root resorptions that are specific to the orthodontic practice.

## RESUMO

As reabsorções radiculares “em plano” ou “em corte” são características do ameloblastoma e foram consideradas patognomônicas dessa lesão, diferenciando-o do cisto ósseo simples, queratocisto odontogênico e do cisto nasopalatino - nos quais elas estão ausentes, nos dentes envolvidos. Apesar dessa frequência elevadíssima e importante no diagnóstico diferencial, não se pode dispensar o exame microscópico, para um diagnóstico definitivo, no planejamento terapêutico do caso. Elaborou-se uma hipótese com seis passos para explicar o mecanismo pelo qual o ameloblastoma promove essa típica reabsorção radicular peculiar da lesão: ser uma neoplasia epitelial benigna sem cápsula fibrosa, formada por ilhotas e cordões epiteliais que imitam a lâmina dentária e invadem os tecidos vizinhos, liberando mediadores (IL-1, EGF) da reabsorção óssea e dentária. Essa hipótese ajuda a explicar mais uma das causas de reabsorções dentárias que podem estar presentes nas análises das documentações ortodônticas, ajudando a diferenciar as reabsorções radiculares próprias da prática ortodôntica.

It may seem incredible, or even unimaginable, that certain issues have not been properly studied and explained in the literature. One of these issues is the real and practical meaning of the presence or absence of resorptions when certain lesions are close to tooth roots or involve them. 

Up to 2019, only two studies ^1^ ^,^ ^2^ have focused on the specific importance of knife-edge tooth resorptions as a characteristic and differential sign of ameloblastomas. Resorptions may distinguish ameloblastomas from other lesions, establish them as a cause of root resorptions, and help differentiate the resorptions found in orthodontic records. When reviewing their cases and images, some authors found that tooth resorptions were the most frequent radiographic finding, but did not describe the characteristics of these resorptions.

## THE FOUR MOST FREQUENT CYSTS ASSOCIATED WITH VITAL TEETH: WHY DOES ONLY AMELOBLASTOMA INDUCE ROOT RESORPTIONS, WHEREAS THE OTHERS DO NOT?

Several diseases appear as cystic images that are radiolucent on radiographs or hypodense on CT scans, and that are associated with neighboring teeth in the jaws. These lesions do not originate or have any association with pulp necrosis or dental problems, and the involved teeth are characteristically vital. 

Four of these lesions are more frequent and are often found on images obtained for orthodontic records. They may come as a surprise for orthodontists that should define the clinical management of these cases and interact with other specialists. These four lesions are:

1) simple bone cyst;
2) nasopalatine duct cyst;
3) odontogenic keratocyst;
4) ameloblastoma.

Other lesions are the glandular odontogenic cyst, botryoidal odontogenic cyst, calcifying odontogenic cyst (Gorlin cysts), odontogenic fibroma, odontogenic myxoma, dentinogenic ghost cell tumor, central giant cell granuloma and other, rarer lesions. In addition to these benign entities, there are also intraosseous malignancies that, in their initial stage, may mimic cysts in vital teeth, such as the clear cell odontogenic carcinoma, spindle cell odontogenic carcinoma and intraosseous epidermoid carcinoma. 

All these conditions require a biopsy to establish a definitive diagnosis. For clinical convenience, orthodontists and other specialists should refer their patients to an oral and maxillofacial surgeon for diagnostic procedures. 

**Figure 1:**
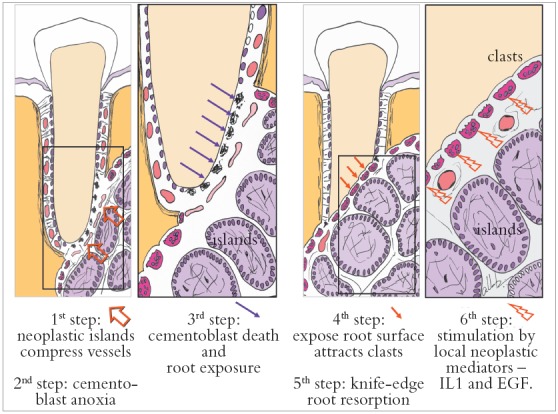
Six steps to explain mechanism of knife-edge root resorption induced by ameloblastoma in involved teeth.

**Figure 2 f2:**
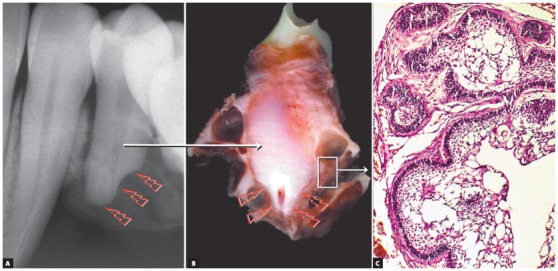
Knife-edge root resorption (arrows) in premolar affected by surgically excised ameloblastoma. In (A), macroscopic radiographic appearance of surgical specimen section; in (B), section for microscopic examination. In (C), epithelial islands with cells that mimic pre-ameloblasts and stellate reticulum of tooth germ and release resorption mediators.

**Figure 3 f3:**
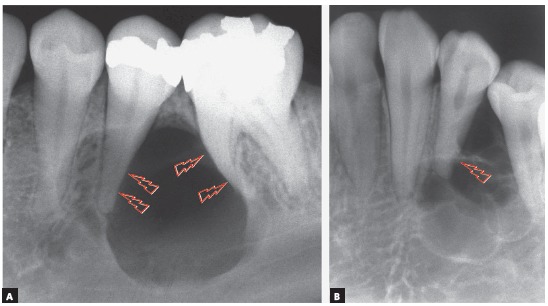
Incipient root resorption in premolars associated with initial stage of ameloblastoma (arrows).

**Figure 4 f4:**
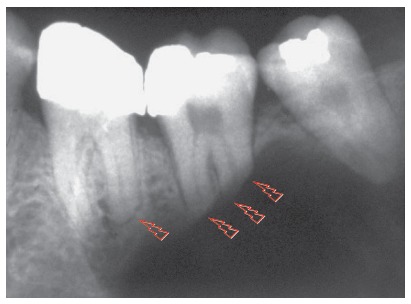
Knife-edge root resorption of involved molars, typical of solid or unilocular ameloblastoma (arrows), regardless of morphological type identified microscopically.

**Figure 5:**
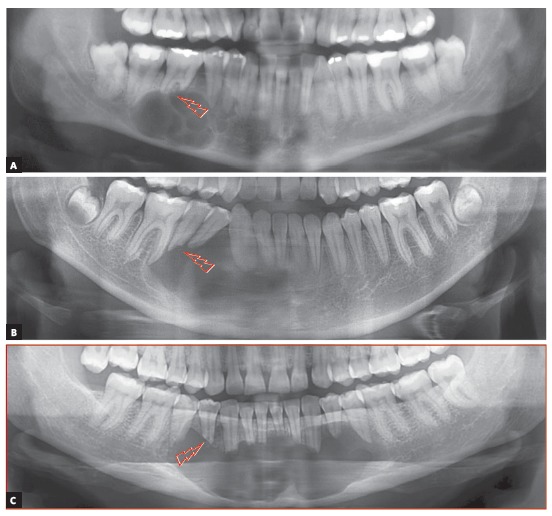
Knife-edge root resorptions of involved teeth, typical of ameloblastoma (arrows), differentiate it from other cysts with similar appearance on imaging studies.

In three of the four most frequent cases of radiolucent or hypodense images of cysts of the jaws associated with neighboring teeth, these teeth remain intact, with no root resorption. These cysts are simple bone cysts, odontogenic keratocysts and nasopalatine duct cysts. However, in ameloblastomas, involved teeth have knife-edge root resorptions (Fig 1 to 5). Why does that occur? 

As a contribution to understanding this phenomenon, this study explores this question and discusses a possible mechanism described in the literature^5^. 

## WHY IS THERE NO ROOT RESORPTION IN TEETH INVOLVED BY ANY OF THESE THREE CYSTS? 

**Figure 6:**
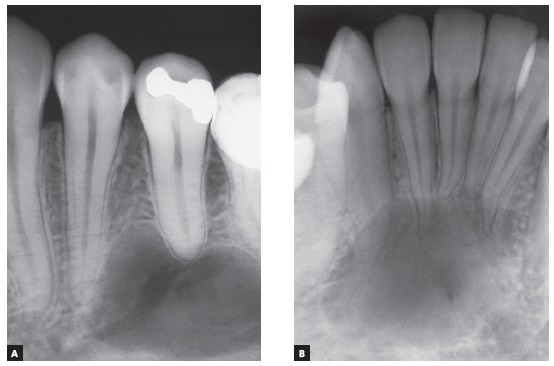
Simple bone cyst: cyst surrounds roots, does not affect lamina dura, periodontal space or root integrity and does not induce root resorptions.

1. The SIMPLE BONE CYST, also known as traumatic or hemorrhagic bone cyst, is the first hypothesis raised for the diagnosis of a radiolucent periapical lesion of mandibular premolars or molars. It may also be found in the region of mandibular incisors or molars, as the mandible is its preferred site, and it may also affect other bones in the body^6^ (Figs 6 to [Fig f7] [Fig f8] 9).

**Figure 7 f7:**
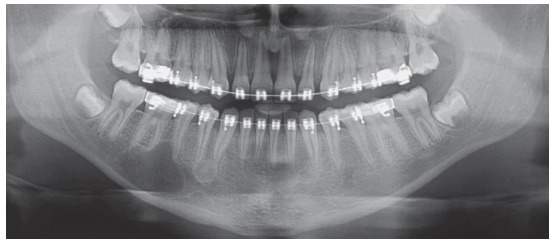
Simple bone cyst surrounds roots as it slowly grows, does not affect lamina dura or periodontal space, nor induces root resorptions. In several cases, cyst originates or develops during orthodontic treatment.

**Figure 8 f8:**
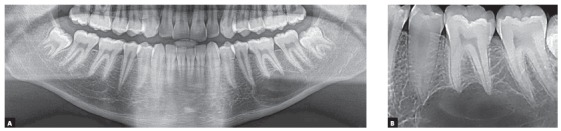
Simple bone cyst surrounds roots, does not affect lamina dura or periodontal space, nor induces root resorptions, regardless of cyst size.

**Figure 9 f9:**
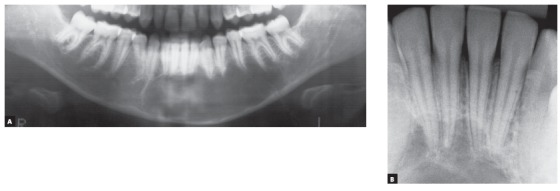
Very large simple bone cyst involving several teeth; roots are surrounded by cyst, but are not resorbed, differently from imaging appearance of ameloblastomas.

» Characteristics: simple bone cysts are cavities with no epithelial lining and whose walls are bare or lined with a thin layer of loose connective tissue. In the cystic cavity, there is air and sometimes a little clear fluid taking up to 20% of its area. Microscopic examination of the fluid obtained by aspiration usually reveals peripheral blood components, because the needle pierces through surrounding tissues to collect it from the cyst.

The presence of air and a diagnosis based on good quality images may establish a definitive diagnosis. At this clinical point, one of the valid treatment options is the filling of the cavity with peripheral venous blood obtained from the patient. The purpose is to form a clot, which is a fundamental structure for bone repair.

The images of simple bone cysts are typically characterized by a continuous and well-defined line that surrounds it and separates it from normal bone (Fig 6 to 9). This cystic line perfectly outlines the involved tooth roots without interrupting the lamina dura; periodontal spaces and root structures are intact, without any tooth resorptions. Involved teeth are vital and not displaced by the presence of this cyst.

Simple bone cysts are asymptomatic, and there is no bulging of the cortical bone, not even when it grows to a few centimeters along its longest axis. An increase in mandible fragility may lead to pathological fractures, even during normal mastication. These cysts grow slowly, and patients do not notice them.

A definitive diagnosis may be established using images and the presence of air in the material collected by aspiration. In addition to a peripheral blood injection into the cystic cavity, another treatment option is to open the cyst and scrape its walls. Then, the cavity is filled with blood to form a clot, which is essential for the formation of new bone and the resolution of the cyst. 

Before filling up the cavity with blood, material for microscopic analysis should be obtained by scraping the cystic bone walls vigorously. After several attempts, the material obtained by repeated curettages may still be minimal, but its analysis may find loose connective tissue free of inflammation and normal bone marrow tissue. If the sample is not sent to the laboratory with a presumptive diagnosis of simple bone cyst, the pathologist may make a descriptive diagnosis of normal bone and marrow tissues. If the pathologist receives information about specimen characteristics on the test request form, the diagnosis in the result report may be “compatible with simple bone cyst”.

The prognosis of simple bone cyst is usually very good, and this cyst does not undergo malignant degeneration or transformation into another type of lesion. This cyst has been called by different terms, in an attempt to associate it with probable causes, such as traumatic bone cyst, hemorrhagic bone cyst or solitary bone cyst. The causes of simple bone cysts are unknown, but some possibilities are local trauma and hemorrhages without the appropriate formation of a blood clot, necessary to scaffold bone repair. Another possible cause is intraosseous venous obstruction, which may trigger resorption in the region.

In addition to treatments that include aspiration, filling with peripheral venous blood and curettage followed by blood clot formation, another suggested approach is imaging control. As it is a cyst typically found in young patients and rarely in people older than 40 years, it may resolve without interventions in many cases. However, mandibular fragility is inevitable during the time the cyst is present, and, therefore, treatment is recommended.

The teeth associated with simple bone cysts are vital. No endodontic treatment should be conducted, as it would not contribute with the resolution of the cyst. However, patients often present with one or more teeth already endodontically treated, indications of a diagnostic error and inadequate clinical management.

» Mechanism that explains the absence of root resorptions: the formation of simple bone cysts is very slow and does not involve any capsule or epithelium; it gradually reabsorbs bone trabeculae, respecting cortical bone, without affecting the periodontal ligament or the pulp (Figs 6 to [Fig f7] [Fig f8] 9). The cementoblasts that protect the roots from resorption are preserved. This cyst grows gradually and does not displace the roots or expand the cortical bone. The reason why there is no tooth resorption in cases of simple bone cysts is the cyst’s slow formation during bone resorption and the absence of compression applied to neighboring structures, as there is no capsule or cystic wall.

**Figure 10 f10:**
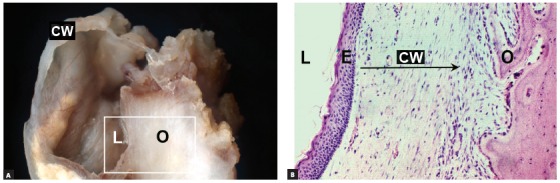
Surgical specimen (A) and microscopic appearance (B) of odontogenic keratocyst, which has a very thin cyst wall (CW), delicate epithelial lining (E) with gradual accumulation of keratin in its lumen (L), which preserves structure of roots involved, without root resorptions; however, bone resorption (O) is slow and continuous.

2. THE ODONTOGENIC KERATOCYST arises from epithelial islands and cords or epithelial rests of the dental lamina randomly distributed in the bones of the jaws, including the periodontal ligament and periapical region (Figs 10 to [Fig f11] [Fig f12] 13). Up to 1% of all apical periodontal cysts may be odontogenic keratocysts and, in these cases, the teeth involved are vital and have no root resorptions.

**Figure 11 f11:**
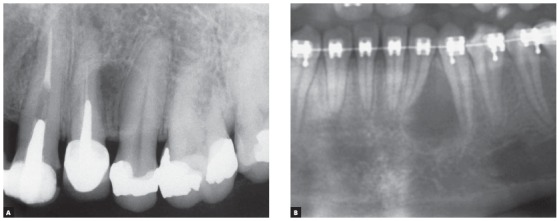
Two initial phases of odontogenic keratocyst, without knife-edge root resorptions (A, B). Cyst did not affect pulp vitality of teeth involved, despite attempts at endodontic treatment. Preserved roots in orthodontic patient (B) are divergent.

**Figure 12 f12:**
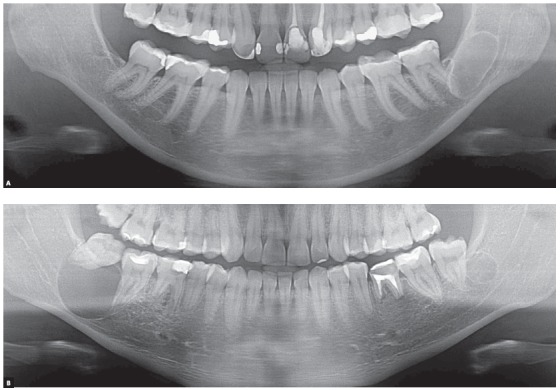
Odontogenic keratocyst involves mandibular molars. In (A), no root resorption is seen, despite neighboring cyst. In (B), two odontogenic keratocysts involving teeth, but cysts do not induce root resorption, which helps make a differential diagnosis with ameloblastoma.

**Figure 13 f13:**
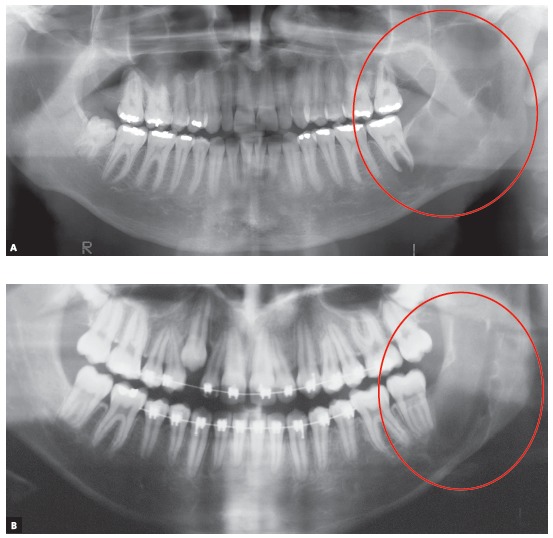
well-developed odontogenic keratocyst in left mandible involves second molars, but there are no root resorptions; appearance is similar to images of ameloblastomas. Absence of root resorption suggest odontogenic keratocyst as first hypothesis in differential and presumptive diagnosis.

» Characteristics: odontogenic keratocysts are asymptomatic and do not rupture or compress the neurovascular bundles that enter the pulp during their growth. For some years, odontogenic keratocysts were incorrectly seen as neoplasms, but recent WHO publications^6^ have again classified them as an odontogenic cyst. During those years, they were also called keratocystic odontogenic tumor.

Odontogenic keratocysts have a high recurrence rate and may give rise to an ameloblastoma on their wall in rare cases and, in even rarer cases, to malignant neoplasms, such as carcinomas. Despite these characteristics, the prognosis for odontogenic keratocysts is good when they are fully excised and followed up postoperatively.

Multiple odontogenic keratocysts are seen in 5% of the patients (Fig 13B), possibly as part of the multiple keratocyst syndrome, also known as nevoid basal cell carcinoma syndrome and Gorlin-Goltz syndrome^6^. In addition to the multiple odontogenic keratocysts, the syndrome may include basal cell carcinomas of the skin and several skeletal anomalies, such as the increase of head circumference, clavicular hypoplasia, spina bifida and bifid ribs. The cause of this syndrome is the deletion of a tumor suppressor gene on chromosome 9.

A definitive diagnosis of odontogenic keratocyst should be made microscopically. The removal of small odontogenic keratocysts that mimic chronic periapical lesions should be considered, as well as an excisional biopsy for microscopic examination.

The surgical removal of an odontogenic keratocyst may affect the mechanical integrity of the neurovascular bundles that enter the pulp, but not in all cases. Therefore, the advantages of endodontic treatment before surgery should be carefully evaluated, as, in some cases, this may be unnecessary, and pulp vitality may be preserved. If the neurovascular bundle is affected, endodontic treatment may be conducted at the time of surgery or immediately after it, without long delay to avoid tooth discoloration.

Endodontists are the professionals that conduct most endodontic surgeries, and their surgical training is sufficient to qualify them technically for this approach to the treatment of odontogenic keratocysts in periapical regions. However, this is not always an appropriate clinical or professional option for several reasons, and the patient may be referred to an oral and maxillofacial surgeon.

» Mechanism that explains the absence of root resorptions: odontogenic keratocysts grow by proliferation of an extremely delicate epithelium that is 5 to 10 cells thick, has no epithelial projections and forms the lining of an extremely thin fibrous capsule, which complicates its en-bloc resection. The keratin layers that fill its lumen form very slowly, with very little compression on neighboring tissues, such as the periodontal ligament. Therefore, cementoblasts are spared, and the root surface is not exposed, which would, otherwise, lead to resorption.^8^ ^-^ ^11^

Odontogenic keratocysts are usually free of inflammation and the numerous mediators that promote tooth and bone resorption. The growth of odontogenic keratocysts is primarily based on progressive epithelial proliferation in some areas, which gives them a wavy appearance similar to that of ameloblastomas. Root resorptions, in these cases, tend to be very slow because there is no inflammation as a source of local mediators (Figs 10 to [Fig f11] [Fig f12] 13).

**Figure 14:**
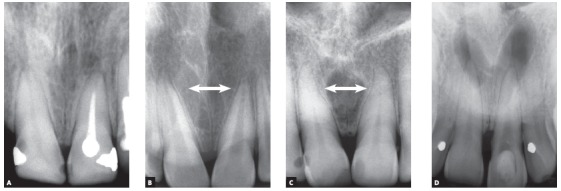
Nasopalatine duct cyst: growth, demonstrated in (A) and (D), with preservation of roots, separation between them in (B) and (C) and convergence of crowns. Cyst is always found in anterior midline, between maxillary central incisors. Lamina dura and periodontal space are not affected because of very slow growth, which prevents root resorption.

3. The NASOPALATINE DUCT CYST is the first diagnostic hypothesis when the cyst is in the midline between the maxillary central incisors (Fig 14). A nasopalatine duct cyst should be suspected initially when apical periodontal cysts are found affecting vital maxillary central incisors. 

» Characteristics: Nasopalatine duct cysts originate in the incisive canal from epithelial remnants of the nasopalatine duct, in the shape of cords and islands. This nonodontogenic developmental cyst, whose triggering factors are unknown, affects the maxillary preferentially^7^. It grows laterally, symmetrically, and it may appear heart-shaped in the anterior maxillary midline when the anterior nasal spine is superimposed (Fig 14). Its lateral growth may lead to a confusion with apical periodontal cysts of maxillary central incisors, but tooth vitality rules out this diagnosis and confirms the presence of a nasopalatine duct cyst.

Maxillary central incisors do not undergo resorption in cases of nasopalatine duct cyst (Fig 14), but their roots may be displaced and diverge, while their crowns converge, and the teeth gradually move along the midline, which may slowly lead to dental crowding. 

The involved maxillary central incisors are vital in the cases of nasopalatine duct cyst. If the patient has any complaint, it is usually because of crown movement and of a salty taste perceived with the tongue right behind the maxillary central incisors. This taste indicates that cyst secretions or fluids are being discretely drained through the periodontal space.

The treatment of nasopalatine duct cysts is surgical, and their prognosis is very good, without recurrences or transformation into another type of lesion. Most cases do not require endodontic treatment of the maxillary central incisors, which remain vital. In a few cases, the neurovascular bundle of the pulp may be affected because of transoperative events, and endodontic treatment may be required at the time of surgery or immediately after it. 

» Mechanism that explains the absence of root resorptions: nasopalatine duct cysts grow very slowly. Therefore, there is enough time for the neighboring central teeth to move and migrate distally and for their roots to diverge, while their crowns converge. The periodontal ligament and the cementoblasts that protect teeth from resorptions are preserved, and root resorptions do not occur in the teeth neighboring the nasopalatine duct cyst.

## WHY DO AMELOBLASTOMAS INVOLVING TEETH HAVE KNIFE-EDGE ROOT RESORPTIONS? 

AMELOBLASTOMAS may mimic periapical lesions when small, but may also mimic a simple bone cyst, an odontogenic keratocyst (Figs 1 to [Fig f2] [Fig f3] [Fig f4] 5), or even although less often, a nasopalatine duct cyst, because this cyst hardly ever affects the anterior maxilla. Ameloblastomas in the periapical region or lateral to tooth roots tend to have a soap-bubble appearance because of their multilocular growth. 

Characteristics: Ameloblastomas and their variations are benign but aggressive odontogenic tumors, as they are invasive and recurrent. Although they are asymptomatic, they may silently resorb teeth, but rarely displace them. Their detection while small or medium-sized, measuring up to 3 cm in diameter, is usually accidental, on images obtained for other purposes (Figs 1 to [Fig f2] [Fig f3] [Fig f4] 5), such as orthodontic records. Ameloblastomas, when large, may lead to tooth mobility and swelling.

Ameloblastomas and odontogenic keratocysts may be very similar,^123^ and only a microscopic examination may distinguish them definitely. Ameloblastomas may also be similar to simple bone cysts in some cases. An important differential sign is the root resorption found in the teeth affected by ameloblastomas, and not, in contrast, in other cysts, such as odontogenic keratocysts.

Martins^5^ studied 50 cases of ameloblastoma and found that, if the roots associated with the cystic-like tumor are resorbed, the chance of a diagnosis of ameloblastoma is so high that resorption may be considered a pathognomonic sign (Figs 1 to [Fig f2] [Fig f3] [Fig f4] 5). If there is no associated root resorption, it is very probably an odontogenic keratocyst or another lesion. Root resorptions in cases of ameloblastomas appear as a knife edge that forms an angle with the long axis of the tooth and touches the tumor tangentially (Figs 1 to [Fig f2] [Fig f3] [Fig f4] 5). Despite the very high frequency and importance of these characteristics for a differential diagnosis, a microscopic examination should not be discarded before defining the diagnosis and treatment plan for these cases (Fig 2).

Ameloblastomas have a high recurrence rate, and their excision requires a large safety margin to avoid recurrences: in such cases, the involved teeth have to be removed together with the surgical specimen. Such an important decision should be made after an incisional biopsy is obtained, because images of other lesions may mimic an ameloblastoma, despite the typical characteristic of root resorptions. As root resorptions are a typical differential sign, their presence may be very helpful when making the diagnosis and planning the treatment to be followed.

Orthodontists with cases of these clinical tumors should immediately make the wise decision to share it and refer the patient to a qualified professional, usually an oral and maxillofacial surgeon (Figs 7, 11 to 13). These surgeons will arrange for a biopsy and treat the ameloblastoma in an extensive surgical intervention, conducted in a hospital.

» Mechanism that explains the presence of root resorptions in ameloblastomas: the following hypothesis has been made to explain the mechanism of root resorption in ameloblastomas (Figs 1 to 2):

1) The epithelial islands and cords in the neoplastic mass of the ameloblastoma move towards the roots and compress the vessels in the periodontal ligament.
2) Hypoxia, later followed by anoxia, leads to the death of cementoblasts, the cells responsible for preserving root integrity, because of the lack of receptors for local and systemic mediators of bone resorption on their cell membranes.
3) The death of odontoblasts exposes the mineralized structure of the root, which, because the periodontal ligament is 0.25-mm thick in average, induces chemotaxis of the abundant clasts in the periodontal region.
4) Clasts on the exposed root surface are juxtaposed to the neoplastic epithelial islands and cords in a parallel or palisade pattern and initiate root resorption.
5) Resorption tends to be uniform or regular, parallel to the interface with neoplastic epithelial islands closer to the root, and the set of clasts generates a regular surface, which appears as a knife-edge root resorption on imaging studies.
6) Resorption continues because of the release of bone or tooth resorption mediators by the ameloblastoma, such as epidermal growth factor (EGF) and interleukin-1 (IL-1). Ameloblastomas are epithelial odontogenic tumors that release EGF and other mediators that induce resorption, such as interleukins^13^ ^,^ ^14^ ^,^ ^15^. These tumors arise from rests of the dental lamina and mimic its infiltrative nature and growth rate in neighboring tissues, without forming a fibrous capsule, typical of benign tumors. Ameloblastoma growth and invasiveness may lead to cementoblast death, as the tumor compresses the periodontal ligament vessels. Without cementoblasts in the root, osteoclasts are attracted to the mineralized surface and are stimulated to resorb it by the presence of high concentrations of neoplastic mediators, such as EGF and interleukins (Figs 1 and 2).

## FINAL CONSIDERATIONS

Knife-edge root resorptions are pathognomic for ameloblastomas (Figs 1 to [Fig f2] [Fig f3] [Fig f4] 5). In simple bone cysts, odontogenic keratocysts and nasopalatine duct cysts, the involved teeth do not have root resorptions (Figs 6 to [Fig f7] [Fig f8] [Fig f9] [Fig f10] [Fig f11] [Fig f12] [Fig f13] 14), which establishes an important differential sign for these cysts when their other clinical and imaging findings are similar to those of ameloblastomas. Despite the very high frequency and importance of these characteristics for a differential diagnosis, a microscopic examination should not be discarded before making a definitive diagnosis and planning the treatment of these cases.

A six-step hypothesis of the progression of ameloblastomas was presented here as a contribution to understanding root resorptions associated with this tumor. This progression is the result of the peculiar characteristics of ameloblastomas, which are benign epithelial tumors without a fibrous capsule and formed by epithelial islands and cords that mimic the dental lamina, invade neighboring tissues and release mediators (IL-1, EGF) of bone and tooth resorption (Figs 1 and 2). In addition to their importance in the differential diagnosis of similar lesions of the jaws, knife-edge root resorptions in cases of ameloblastoma may be one more explanation for root resorptions found on the images of orthodontic records (Figs 7, 11 and 13), and may, thus, help differentiate resorptions that are specific to the orthodontic practice.
